# Supplementary figures and images for: Loss of meiotic double strand breaks triggers recruitment of recombination-independent pro-crossover factors in C. elegans spermatogenesis
Source: PLoS Genet. 2025 Oct 22;21(10):e1011763. doi: 10.1371/journal.pgen.1011763 (PMC12561964; doi:10.1371/journal.pgen.1011763)

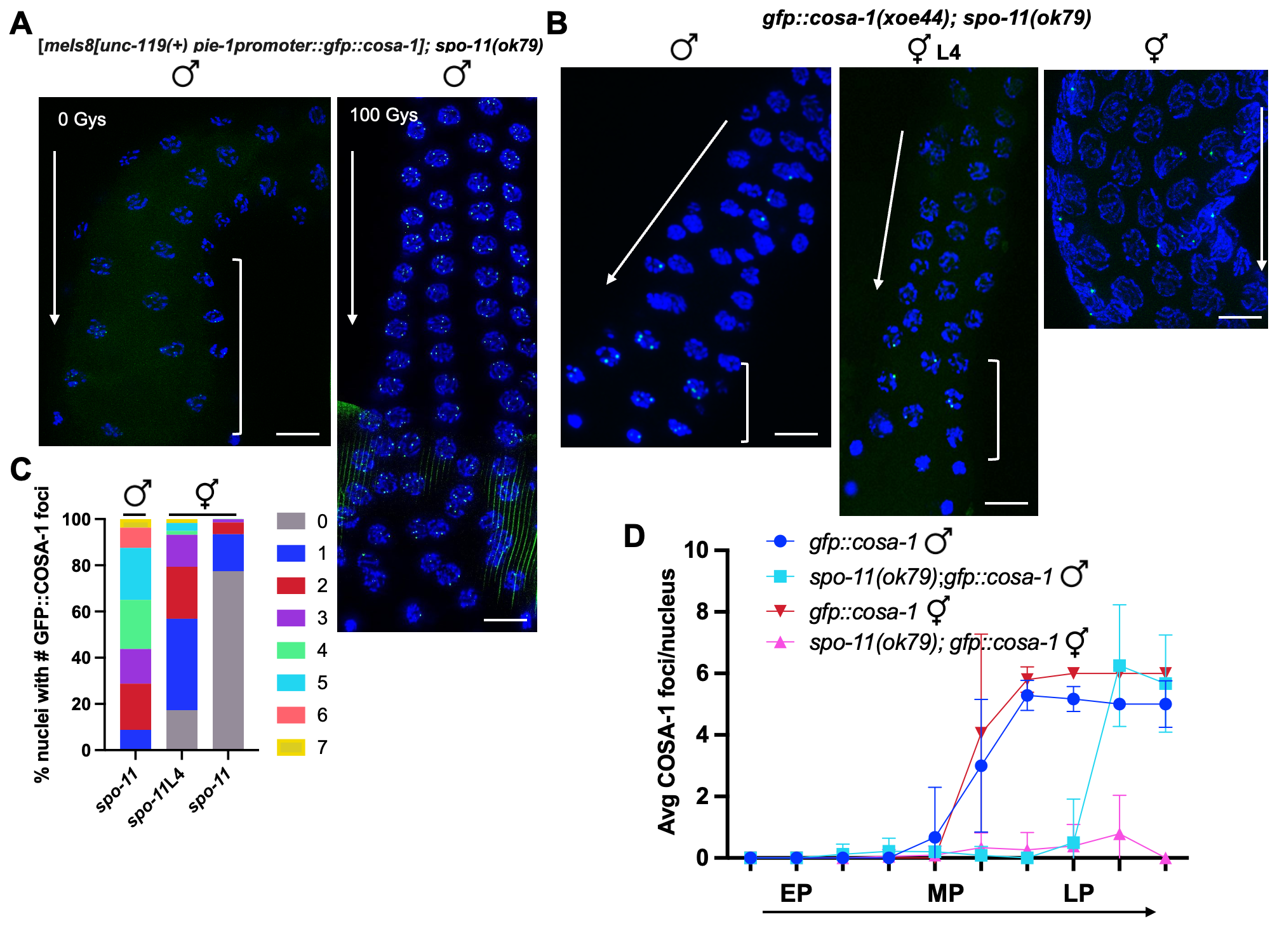

Supplement: S1 Fig — (A) Images of pachytene/condensation zones of male [meIs8[unc-119(+) pie-1promoter::GFP::cosa-1]; spo-11(ok79) germ lines imaged for GFP::COSA-1 fluorescence (green) and counterstained with DAPI (blue) in the absence (0Gys) or presence (100Gys) of IR. Bracketed region denotes faint GFP::COSA-1 foci in the [meIs8[unc-119(+) pie-1promoter::GFP::cosa-1]; spo-11(ok79) mutant in the absence of IR. (B) Image of pachytene/condensation zones of GFP::cosa-1(xoe44); spo-11(ok79) males, L4 hermaphrodites undergoing spermatogenesis and adult hermaphrodites (24h post L4). White arrow denotes direction of meiotic progression. Bracket denotes region of germ line with GFP::COSA-1 foci. Scale bar 10 μm. (C) Stacked bar graph showing percent nuclei with indicated numbers of GFP::COSA-1 foci in GFP::cosa-1(xoe44); spo-11(ok79) males, L4 and adult hermaphrodites. Three germ lines were examined; 80 nuclei were scored in males, 58 nuclei were scored in L4 hermaphrodites and 98 nuclei were scored in adult hermaphrodites. Number of GFP::COSA-1 foci: grey = 0; blue = 1; red = 2; purple = 3; neon green = 4; cyan = 5; rose = 6; gold = 7. (D) Mean number and S.D. of GFP::COSA-1 as scored through the germ line from early pachytene (EP), mid pachytene (MP) and late pachytene (LP) from a minimum of 3 germ lines. (TIF) [file pgen.1011763.s001.tif]

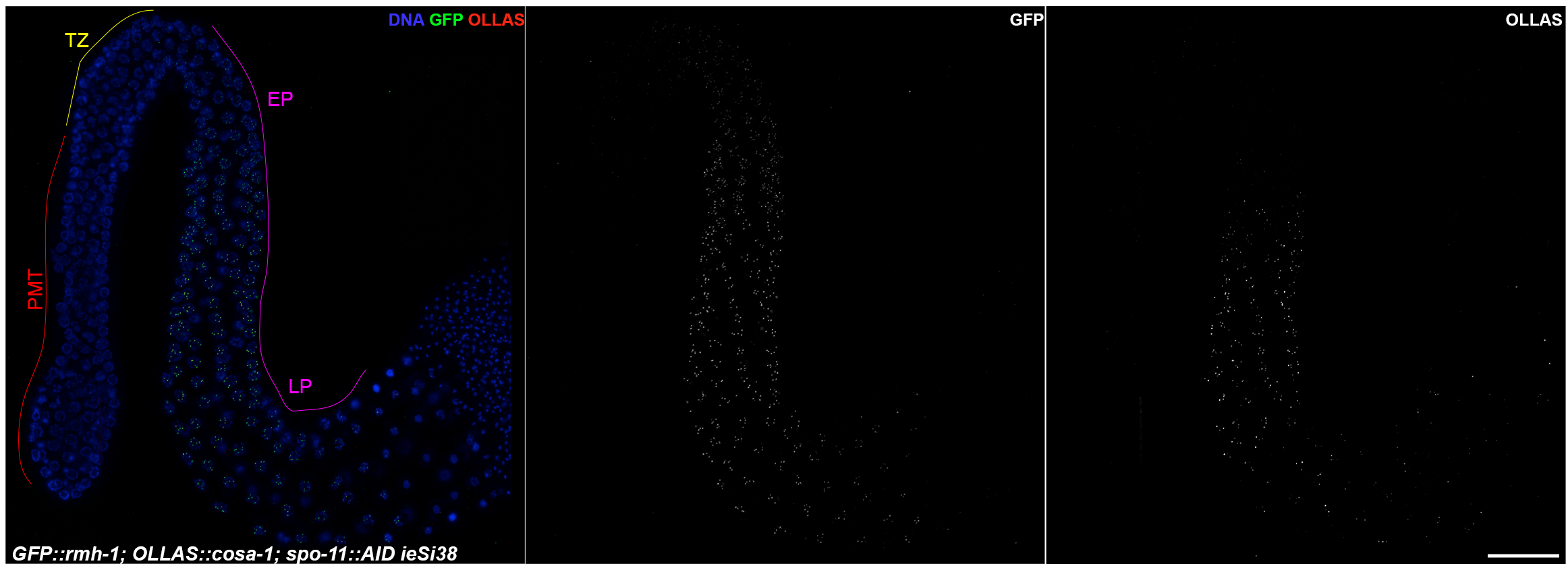

Supplement: S2 Fig — Whole-mount male germ line of the indicated genotype immunolabeled for GFP::RMH-1 (green) and OLLAS::COSA-1 (red) counterstained with DAPI (blue), showing abundant RMH-1 foci formation in early pachytene and recruitment to presumptive CO sites together with COSA-1 in late pachytene cells. PMT: pre-meiotic tip; TZ: Transition Zone; EP: Early Pachytene; LP: Late Pachytene. Scale bar 20 μm. (TIF) [file pgen.1011763.s002.tif]

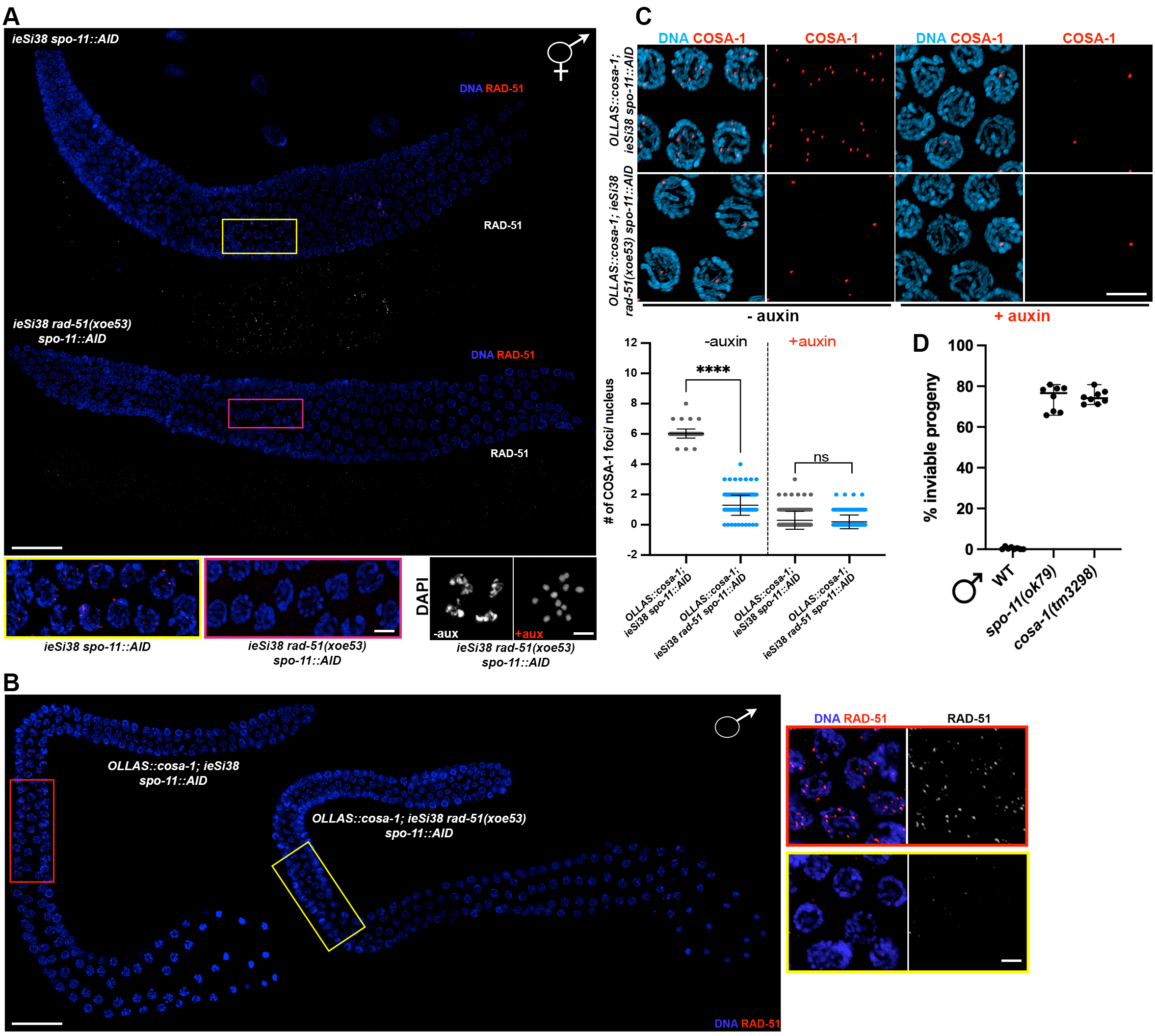

Supplement: S3 Fig — (A) Top: Whole-mount hermaphrodite gonads of the indicated genetic backgrounds immunoassayed for RAD-51 (red) and counterstained with DAPI (blue). Scale bar 20 μm. Color-coded insets (bottom left) show magnified early-mid pachytene regions to show no detectable RAD-51 foci in the OLLAS::cosa-1; spo-11::AID rad-51(xoe53) ieSi38 worms compared to OLLAS::cosa-1; spo-11::AID ieSi38 controls. Scale bar 20 μm. Bottom right: representative images of DAPI-stained diakinesis nuclei depicting aberrant chromatin bodies in the OLLAS::cosa-1; spo-11::AID rad-51(xoe53) ieSi38 (-auxin) whose formation is suppressed upon SPO-11 depletion (+auxin). Scale bar 2 μm. (B) Left: whole-mount male gonads of the indicated genetic backgrounds immunolabeled for RAD-51 (red) and counterstained for DAPI (blue). Scale bar 20 μm. Right: magnified color-coded insets showing early-pachytene nuclei in control and rad-51(xoe53) mutant worms showing no detectable RAD-51 foci. Scale bar 2 μm. (C) Top: late-pachytene oocytes in the indicated genetic backgrounds and exposure conditions to auxin immunolabeled for OLLAS::COSA-1 (red) and counterstained for DAPI (cyan). Scale bar 5 μm. Bottom: quantification of OLLAS::COSA-1 foci number in the same genotypes and exposure conditions to auxin. Bars show mean with S.D. and asterisks denote statistical significance assessed by Kolmogorov-Smirnov test (****p < 0.0001, ns = not significant). The number of nuclei analyzed in controls and rad-51(xoe53) are 122–172 (-auxin) and 130–160 (+auxin). (D) Percent inviability of fog-2(q71) progeny sired by WT (N2), spo-11(ok79), and cosa-1(tm3298) males. Mean and 95% confidence intervals are shown from a total of 8 matings. No statistical difference between spo-11 and cosa-1 was observed by Mann-Whitney. (TIF) [file pgen.1011763.s003.tif]

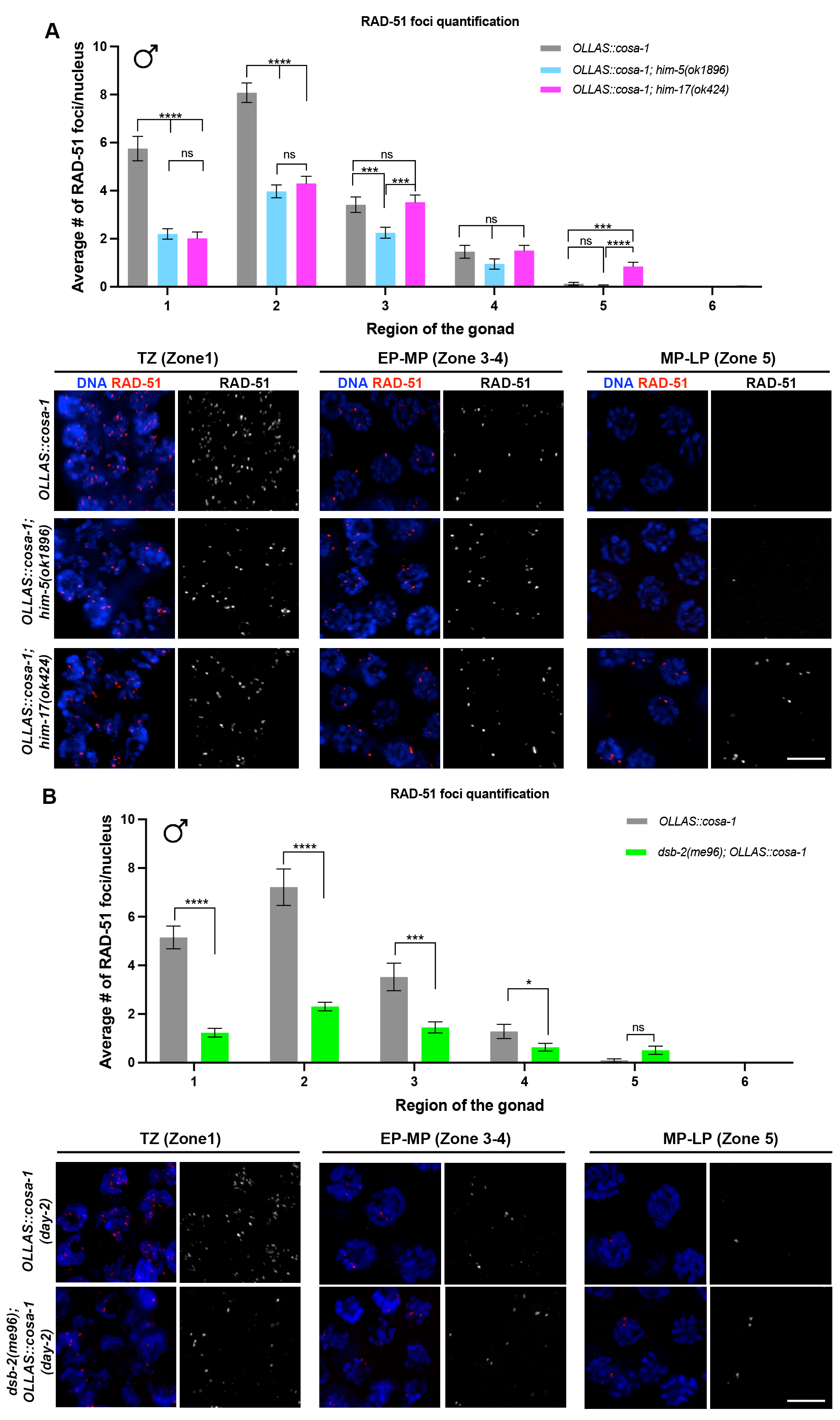

Supplement: S4 Fig — (A) Quantification (top) and representative images (bottom) of nuclei from controls, him-5 and him-17 mutants at the indicated stages immunoassayed for RAD-51 (red) and counterstained with DAPI (blue). Scale bar 5 μm. The number of nuclei analyzed from zone 1–6 was: OLLAS::cosa-1 (86 – 63 – 53 – 48 – 47 – 40), OLLAS::cosa-1; him-5(ok1896) (129 – 70 – 56 – 59 – 61 – 65), OLLAS::cosa-1; him-17(ok424) (104 – 66 – 44 – 53 – 51 – 62). (B) Quantification (top) and representative images (bottom) of nuclei from aged (48h post L4) controls and dsb-2 mutants at the indicated stages immunoassayed for RAD-51 (red) and counterstained with DAPI (blue). Scale bar 5 μm. The number of nuclei analyzed from zone 1–6 was: OLLAS::cosa-1 (54 – 28 – 21 – 21 – 21 – 25), OLLAS::cosa-1; dsb-2(me96) (77 – 72 – 49 – 44 – 43 – 52). (TIF) [file pgen.1011763.s004.tif]

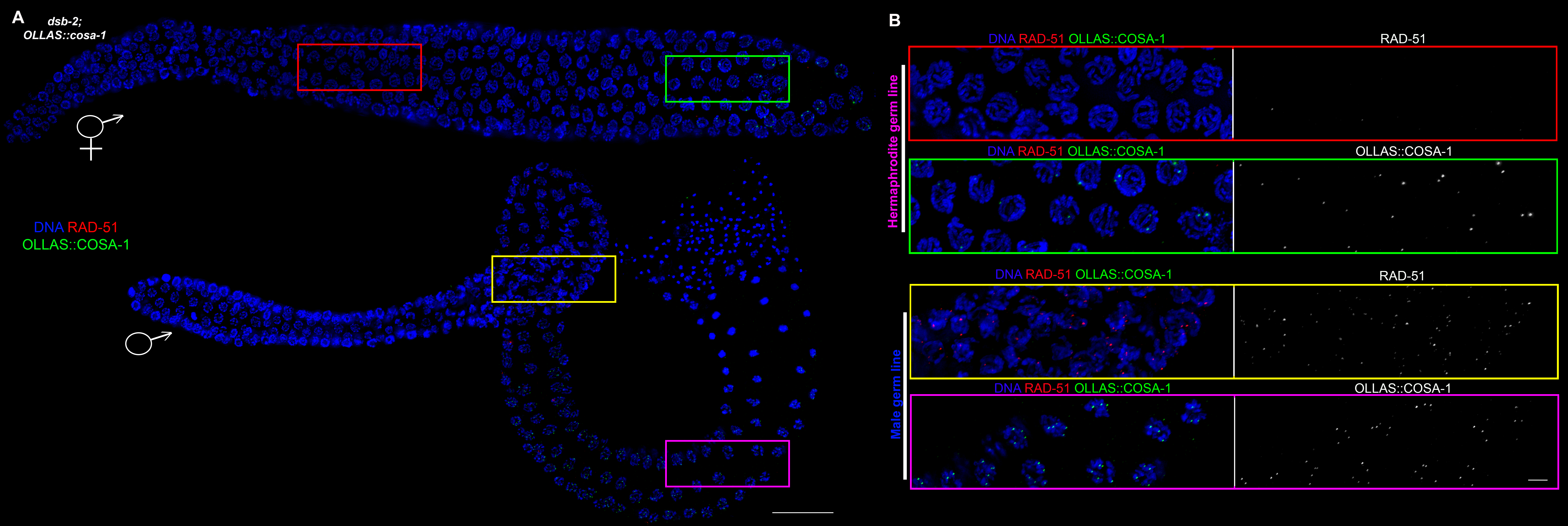

Supplement: S5 Fig — (A) Whole-mount hermaphrodite and male germ lines dissected 48h post-L4 and immunostained for RAD-51 (red)/OLLAS::COSA-1 (green) and counterstained with DAPI (blue). Scale bar 20 μm. (B) Color-coded insets showing magnified early and late pachytene cells from the indicated sexes depicting more robust recruitment of RAD-51 and COSA-1 in males versus hermaphrodites. Scale bar 5 μm. (TIF) [file pgen.1011763.s005.tif]

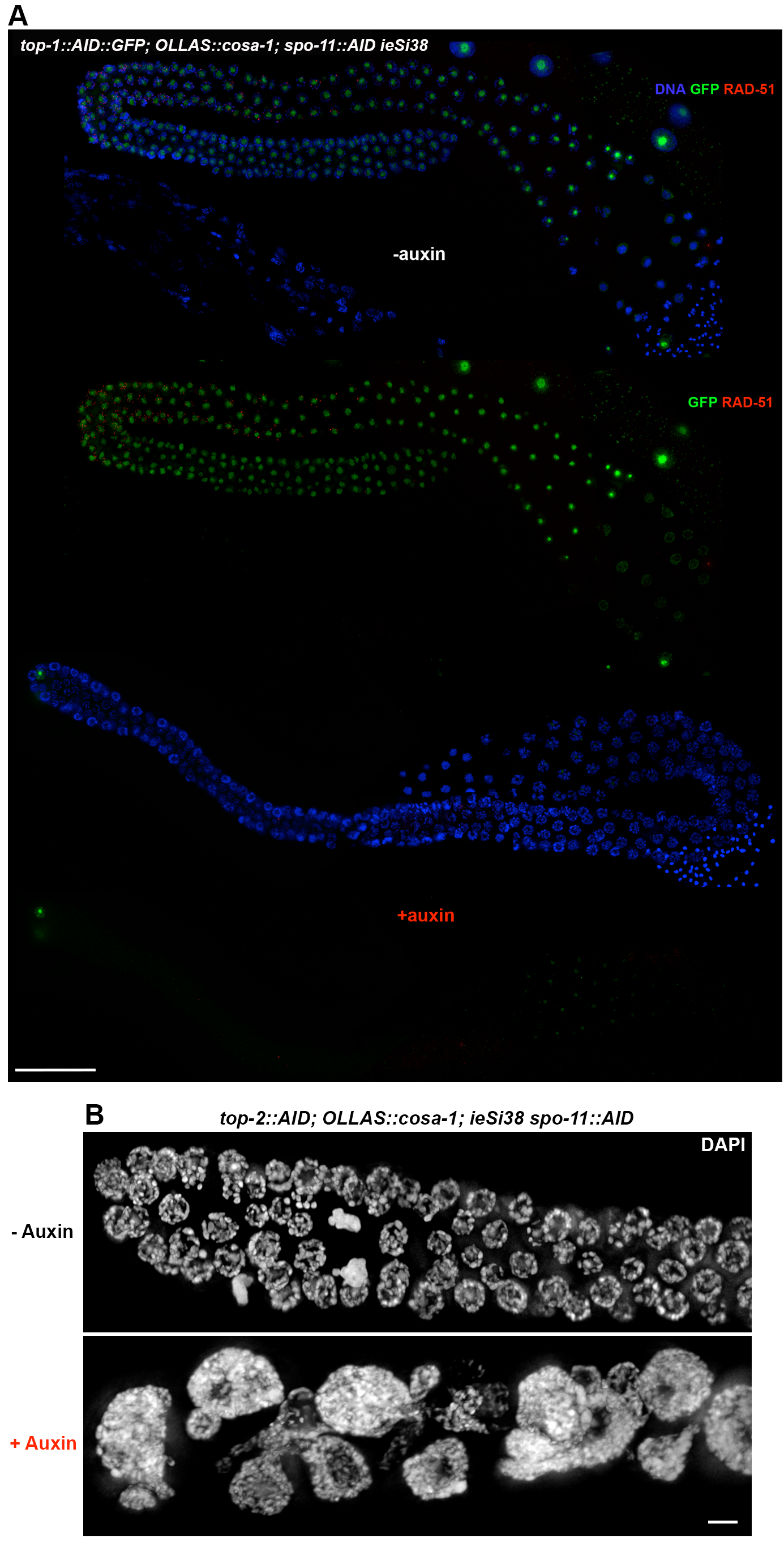

Supplement: S6 Fig — (A) Whole-mount male germ lines from the indicated genotype immunoassayed for TOP-1::AID::GFP (green), RAD-51 (red) and counterstained with DAPI (blue). TOP-1 is efficiently depleted upon 24h exposure to auxin. Scale bar 20 μm. (B) DAPI staining of the distal tip from top-2::AID animals before and after exposure to auxin. Note mitotic catastrophe elicited by TOP-2 depletion, as indicated by enlarged nuclei, micronuclei and chromatin bridges. Scale bar 2 μm. (TIF) [file pgen.1011763.s006.tif]

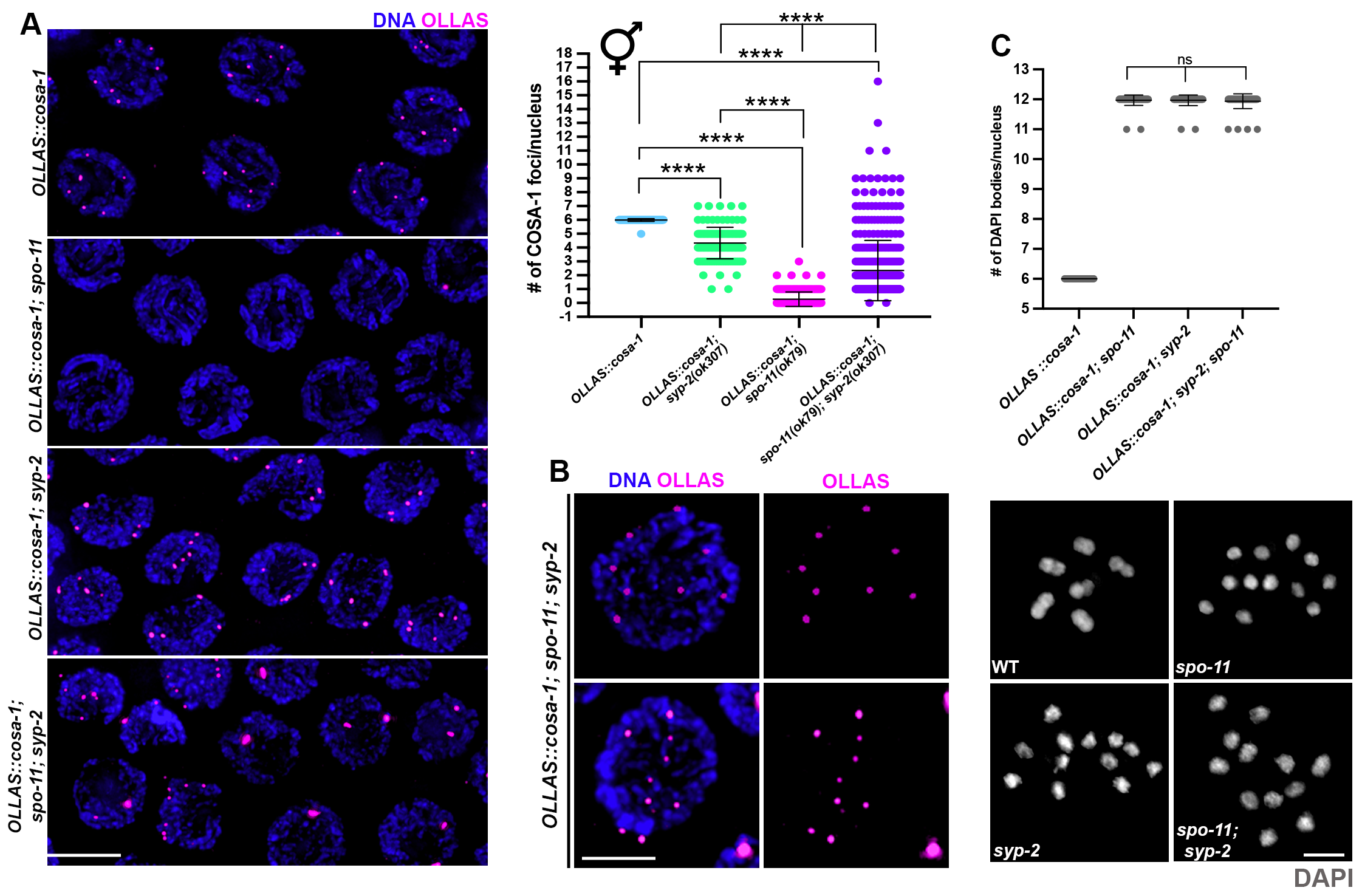

Supplement: S7 Fig — (A) Left: representative images of late pachytene oocytes of the indicated genotypes stained with OLLAS (COSA-1; magenta) and counterstained with DAPI (blue). Scale bar 2 μm. Right: quantification of OLLAS::COSA-1 foci in the indicated mutant backgrounds. The number of nuclei analyzed was: OLLAS::cosa-1 (116), OLLAS::cosa-1; syp-2(ok307) (123), OLLAS::cosa-1; spo-11(ok79) (180), OLLAS::cosa-1; spo-11(ok79); syp-2(ok307) (426). Bars indicate S.D. and asterisks denote statistical significance assessed by Kolmogorov-Smirnov test (****p < 0.0001). (B) High magnification of two examples of late pachytene nuclei from OLLAS::cosa-1; spo-11(ok79); syp-2(ok307) mutants displaying extensive OLLAS::COSA-1 foci. Scale bar 2 μm. (C) Quantification (top) and representative images (bottom) of DAPI bodies in Diakinesis nuclei of the indicated genotypes. Scale bar 2 μm. Bars depict S.D. and statistical comparison by T test indicates non-significant difference (ns). The number of Diakinesis nuclei scored was: OLLAS::cosa-1 (41), OLLAS::cosa-1; syp-2(ok307) (63), OLLAS::cosa-1; spo-11(ok79) (64), OLLAS::cosa-1; spo-11(ok79); syp-2(ok307) (63). (TIF) [file pgen.1011763.s007.tif]
