# Supplementary material for: Loss of meiotic double strand breaks triggers recruitment of recombination-independent pro-crossover factors in C. elegans spermatogenesis
Source: PLoS Genet. 2025 Oct 22;21(10):e1011763. doi: 10.1371/journal.pgen.1011763 (PMC12561964; doi:10.1371/journal.pgen.1011763)
Supplement: S1 Table — (DOCX) [file pgen.1011763.s008.docx]

**S1 Table: Worm strains**

| Name | Genotype | Source |
| --- | --- | --- |
|  |  |  |
| JEL993 | GFP(glo)::3xflag::cosa-1(xoe44) *III* | Li *et al.*, 2022 |
| JEL1369 | *tra-2(e1095)/dpy-10(e128) unc-4((e120) II;* GFP(glo)::3xflag*::cosa-1(xoe44 )III* | This study |
| JEL1367 | GFP(glo)::3xflag*::cosa-1(xoe44) III; ced-3(ok2734) IV* | This study |
| JEL986 | [meIs8[unc-119(+) pie-1promoter::GFP::cosa-1] II; *spo-11(ok79)/nT1 [unc-?(n754) let-?] (IV;V)* | This study |
| JEL1395 | GFP(glo)::3xflag::cosa-1(xoe44) *III; spo-11(ok79)/nT1 [unc-?(n754) let-?] (IV;V)* | This study |
| AV596 | *cosa-1(tm3298)/qC1[qIs26] III* | Yokoo et al., 2012 |
| CB4108 | *fog-2(q71) V* | CGC |
| ERC84 | *ers56[top-1::degron::GFP] I; ieSi57 [eft-3p::TIR1::mRuby::unc-54 3'UTR + Cbr-unc-119(+)] II* | Morao et al.; 2022 |
| CA1199 | *ieSi38 [sun-1p::TIR1::mRuby::sun-1 3´UTR + Cbr-unc-119(+)] IV* | CGC |
| NSV623 | *OLLAS::cosa-1 III; spo-11::AID::3xFLAG rad-51(xoe53) ieSi38/nT1 (IV;V)* | This study |
| YKM240 | *cdk-2::HA I* | Haversat et al.; 2022 |
| NSV646 | *cdk-2::HA I; OLLAS::cosa-1 III; spo-11::AID::3xFLAG ieSi38 IV* | This study |
| NSV420 | meIs8[unc-119(+) Ppie-1::GFP::cosa-1 *II; OLLAS::cosa-1 III; spo-11::AID::3xFLAG ieSi38 IV* | Hicks et al.; 2022 |
| NSV688 | *rmh-1 [syb9786 (GFP::rmh-1)] I* | This study |
| NSV662 | *GFP::rmh-1 I; OLLAS::cosa-1 III; spo-11::AID ieSi38 IV* | This study |
| NSV97 | *cosa-1 [ddr12(OLLAS::cosa-1)] III* | Janisiw et al.; 2018 |
| NSV138 | *OLLAS::cosa-1 III; him-5(ok1896) V* | Janisiw et al.; 2020 |
| NSV690 | *OLLAS::cosa-1 III; him-17(ok424)/nT1 (IV;V)* | This study |
| NSV694 | *OLLAS::cosa-1 III; dsb-1(we11)/nT1 (IV;V)* | This study |
| NSV700 | *dsb-2(me96); OLLAS::cosa-1* | This study |
| NSV676 | *OLLAS::cosa-1 III; spo-11::AID::3xFLAG ieSi38/nT1; syp-2(ok307)/nT1 (IV;V)* | This study |
| JEL1393 | *top-1::AID::GFP I; OLLAS::cosa-1 III; spo-11::AID::3xFLAG ieSi38 IV* | This study |
| ATG341 | *top-2 [fq43(top-2::AID)] II; ieSi38 IV* | This study |
| NSV658 | *top-2::AID II; OLLAS::cosa-1 III; spo-11::AID::3xFLAG ieSi38 IV* | This study |
