# Supplementary material for: Loss of meiotic double strand breaks triggers recruitment of recombination-independent pro-crossover factors in C. elegans spermatogenesis
Source: PLoS Genet. 2025 Oct 22;21(10):e1011763. doi: 10.1371/journal.pgen.1011763 (PMC12561964; doi:10.1371/journal.pgen.1011763)
Supplement: S2 Table — (DOCX) [file pgen.1011763.s009.docx]

**S2 Table: Antibodies used in this study**

| Antibody | Dilution | Source |
| --- | --- | --- |
| Monoclonal Mouse anti-HA | 1:600 | BioLegend (#901501) |
| Polyclonal Rabbit anti-SYP-1 | 1:1000 | Janisiw et al.; 2020 |
| Polyclonal Rat anti-SYP-1 | 1:200 | Hicks et al.; 2022 |
| Polyclonal Rabbit anti-RAD-51 | 1:3000 | Das et al.; 2022 |
| Polyclonal Rat anti-RAD-51 | 1:500 | Blazickova et al.; 2025 |
| Polyclonal Rabbit anti-OLLAS | 1:1000 | Genscript (#A01658) |
| Monoclonal Mouse anti-GFP | 1:500 | Roche (#11814460001) |
