# Supplementary material for: Loss of meiotic double strand breaks triggers recruitment of recombination-independent pro-crossover factors in C. elegans spermatogenesis
Source: PLoS Genet. 2025 Oct 22;21(10):e1011763. doi: 10.1371/journal.pgen.1011763 (PMC12561964; doi:10.1371/journal.pgen.1011763)
Supplement: Attachment — Submitted filename: Response to reviewers929.docx [file pgen.1011763.s011.docx]

We would like to thank the Reviewers for their overall support of our work and constructive suggestions to improve the manuscript. We provide below a detailed point-to-point answer to their concerns (in red text).

**Guest Editor comments:**

1) I share the questions of reviewers 2 and 3 regarding the altered distribution of COSA-1 in the SPO-11 depleted germ lines. It would be helpful to discuss why more COSA-1 foci would be present and why there is such a broad distribution of COSA-1 foci.

We have added additional data (see S1D Fig) and further discussion on this point (see also response to Reviewer 1, point 2 and Reviewer 3, point 5).

2) Throughout the manuscript and figures TIR is referenced in several different ways (e.g. TIR1, TIR1::mRuby, or not at all) making it a little confusing as to when it is present and whether there are different TIR1 genes present. Please edit for consistency and clarity.

We have replaced “TIR1” with “ieSi38” (psun-1::TIR1::mRuby::sun-1 3’ UTR), carried by all AID strains employed in our study integrated on Chromosome IV, and that corresponds to the original transgene generated by the Dernburg lab (Zhang and Dernburg, 2015).

3) Figure 5. Related to comment 6 from reviewer 3; Provide the genotype for the strain(s) used in Panel C or add the proteins to GFP and OLLAS.

We apologize for overlooking this detail. We have fixed this in the figure (now Fig 4B), which now displays the genotype correctly indicated.

4) Figure 7. Please include the statistics for OLLAS::cosa-1; spa-11::AID TIR -auxin and plus auxin (Panel A) and between OLLAS::cosa-1; spo-11::AID TIR1 (-auxin) and OLLAS::cosa-1; spo-11::AID TIR1 syp-2(ok307) (-auxin) (Panel B).

Statistical comparisons have been included as requested.

5) In the section "SPO-11-independent COSA-1 foci also form independently of Topoisomerase I or II," care is taken to show that TOP-1 is depleted efficiently, but there is no mention of how TOP-2 depletion was monitored. Please include.

Unfortunately, the top-2::AID allele is not tagged with an epitope for detection and thus we relied on a phenotypic readout of TOP-2 depletion. We have included representative images of the mitotic tip from top-2::AID; OLLAS::cosa-1; spo-11::AID ieSi38 before and after auxin exposure, that show clear enlargement of mitotic nuclei as a readout of TOP-2 depletion (S6B Fig).

**Reviewer #1:**

1) Title: The phrase “recombination-independent pro-crossover factors” seems counterintuitive - a pro-crossover factor cannot be independent of recombination (since crossovers are, by definition, recombination events). Perhaps better to state that the recruitment is recombination-independent instead of the factors themselves being recombination-independent. Perhaps: “Loss of meiotic double strand breaks triggers recruitment of pro-crossover factors to chromatin in C. elegans spermatogenesis”

We would rather maintain the Title as is, since recruitment of COSA-1-RMH-1-CDK-2 in spo-11 depleted germ line occurs independently of the only meiotic recombinase present in worms (RAD-51) and as such, it highlights the uniqueness of our findings. All these factors, including MSH-4/5 and the ZHPs protein family, are collectively indicated as ‘pro-CO factors’ even if they do not label uniquely CO events but also early recombination intermediates (such as MSH-4/5 and RMH-1), therefore we believe it is correct to state the title as is.

2) I agree that prior genetic data indicating more double crossover events provides evidence for less robust crossover control in the male. However I am less convinced that the extra COSA-1 focus observed in the male germline is a cytological reflection of less robust crossover control (perhaps I am too influenced by the second part of the study that demonstrates additional recombination-independent substrates for COSA1 arise, particularly in male germlines). This is just a comment - In the end I do not think this is an issue needing resolved for this study, as the extra COSA-1 foci in the male germline is a difference between spermatogenesis/oogenesis worth exploring regardless of whether it corresponds to a future crossover or not. This uncertainty however (in my brain) tends to muddle my thinking of whether the SPO-11+ and spo11 minus COSA-1 phenomena are somehow connected.

The nature of the COSA-1 foci is distinct in wild type vs. the spo-11 mutant. In wild type we observe mostly 5 (or 6) strong COSA-1 foci between mid-late pachytene. In spo-11 we observe a very broad distribution of COSA-1 foci that is observed in late pachytene /condensation zone. We have tried to make this point in the manuscript (see response to Guest Editor #1 above). This is also consistent with the observed viability of male sired progeny in wild type vs. spo-11 (S3D Fig). That being said, we are cognizant of the differences between COSA-1 foci and crossovers. To address this, we have replaced COs with COSA-1 foci throughout the manuscript to be clear that this is what we monitored. However, as the Reviewer states, this does not infringe on the weight of the finding itself.

3) Abstract: “and that there are previously unrecognized lesions or structures at the end of meiotic prophase in spermatocytes that can accumulate CO markers” And, at the end of the Introduction: "Surprisingly, we discovered that COSA-1 and its partners accumulate at the end of meiotic prophase in the absence of DSBs specifically in spermatogenesis." But don’t hermaphrodite germlines show the same lesions, only fewer of them per nucleus? (Supplemental Fig 7 indicates that one to three COSA-1 foci are observed in a substantial fraction of spo-11 hermaphrodite germline nuclei.) If I understand this correctly, the Abstract and Intro should be modified to avoid implying that COSA-1 associated lesions arising due to SPO-11 removal are exclusive to the male germline.

The reviewer is correct that very few COSA-1 foci are present in spo-11-depleted oogenic gonads, and it has been extensively shown in the literature that these arise from RAD-51-mediated invasions of occasional pre-meiotic damage. However, in the male germ lines, we observe a distinct pattern, where almost all nuclei in late pachytene/condensation zone have COSA-1 foci, and while the number is variable there can be as many as 10 foci, something never observed in hermaphrodites. We have included additional quantification of these foci in hermaphrodites, males and L4 hermaphrodites undergoing spermatogenesis – please see Fig 2 and S1 Fig.

4) Currently the study reads almost like it is two separate papers back-to-back. To streamline the narrative, I recommend that data in Figures 1 and 2 be consolidated (the genetic pathways in Figure 2 are not necessary and can be removed). The Results describing experiments in Figures 1 and 2 could maybe also be made more concise. If the first part (addressing COSA-1 foci in SPO-11+ germlines) is shorter, the reader may be more attentive to the distinctive aspects of the second set of major observations (which I think make up the majority of the study).

We have modified according to the Reviewer’s advice.

5) Using COSA-1 as a marker for crossovers and gamma radiation to introduce DNA DSBs, the authors observe that males have a (more or less) linear response in converting increasing #s of DSBs into crossovers. The narrative concludes that males are different from hermaphrodites in this respect, but only shows a gamma radiation dose experiment performed on males, comparing the results to prior hermaphrodite data from a different lab. For maximal confidence in the conclusion that males and hermaphrodites differ in their degree of crossover homeostasis at low and high doses of radiation, hermaphrodites and males should be treated in parallel (optimally exposed to radiation in the same culture dish) and using the same equipment and immunofluorescence conditions. If this kind of rigorous comparison is not possible, the strength of conclusions made (about differences between male and hermaphrodites) should be dialed back in the narrative.

We performed the IR experiment with hermaphrodites at the same time and have now included the data in Fig 2B and 2C. Note that we did not have data for hermaphrodites treated with 50Gys of IR; however, by 10Gys hermaphrodites have mostly six GFP::COSA-1 foci as previously published (Yokoo et al., 2012). This has allowed us to directly compare males and hermaphrodites and observe a statistical difference.

6) Figure 4’s title reads “SPO-11 activity in males is extended throughout prophase.” But the data shown in this figure, suggest (to me) that SPO-11 activity is somewhat restricted to zones 1 and 2, not extended throughout prophase. Even after 2 hours of treatment with auxin to remove SPO-11 activity, RAD-51 foci only substantially diminish in zone 1 and 2 (not 3 and 4). Only after 4 hours of exposure to auxin do we observe substantive diminishment of RAD-51 in the later zones 3 and 4. I suggest changing the title, and perhaps instead focus the title on the surprise finding that SPO11 removal causes recruitment of COSA-1 foci to chromatin.

We have modified the title as recommended.

7) The Results section “SPO-11 depletion in the male germ line leads to a similar abrogation of DSBs as in the hermaphrodite germline” is written clearly, but nevertheless I read it a few times because I was unsure what question the authors were addressing with the experiment. The end of the section states “These results indicate that depletion of SPO-11 leads to reduction of DSBs in a similar pattern to hermaphrodites”, but it is unclear what this experiment was trying to get at in the first place. In other words, was there a different possible outcome that would have suggested something specific about recombination in the male germline? Because this section seems to be an experiment performed without a specific hypothesis in mind, I wonder if this section subheading could be removed and these results streamlined/combined with the following section that reports the interesting observation of [COSA-1 foci induced by SPO-11 removal]? The data on RAD-51 and fertility could perhaps be extracted and constitute an independent Results subsection.

Following the reviewer’ s advice, we have modified this section accordingly.

8) Results subheading: “COSA-1 foci accumulate independently of SPO-11 and RAD51 in late pachytene spermatocytes” – this heading is technically correct but caution to the authors because it might imply that there is a population of SPO-11 and RAD51-independent COSA-1 foci even in normal wild type meiosis. An alternative title could be: “Removal of SPO-11 causes the appearance of COSA-1 foci in late pachytene” (or, “RAD-51-independent COSA-1 foci in late pachytene”, if the authors decide not to extract the RAD-51 data into an independent results subsection).

We agree with the reviewer and have changed the subheading and reordered the data as indicated.

9) In the Results subsection mentioned above (pt. 7), the authors imply that hermaphrodites depleted of SPO-11 have a block in COSA-1 formation, and a prior study is cited. However, data in Figure 7 (to my eyes) indicates that many nuclei in the SPO-11-depleted hermaphrodite germline have one, two, or three COSA-1 foci. This set of hermaphrodite data (COSA-1 foci in SPO-11 depleted hermaphrodite germlines in Figure 7) should be brought to this earlier position of the manuscript and fully discussed for clear comparison between males and hermaphrodites. (It is not clear to me what region of the hermaphrodite gonad these COSA-1 foci-positive nuclei are found, thus I think this also should be indicated, so that the reader can understand how they may or may not connect to the COSA-1 positive nuclei found in SPO-11- depleted male germlines.) The data suggest (to me) that COSA-1 foci are found in some hermaphrodite germline nuclei, although they do not rise to the abundance found in some male germline nuclei. Male germline nuclei in the (spo-11 knockout) hermaphrodite L4, however, seem to exhibit predominantly 1-3 COSA-1 foci, resembling what appears to be the case for hermaphrodite oogenic germline nuclei. If I have understood the data correctly, the statements implying that COSA-1 – positive nuclei are specific to male germline nuclei should be modified (e.g.: “and is specific to spermatogenesis in both males and L4 hermaphrodites.”).

See Response #3 (the mentioned COSA-1 foci in spo-11 hermaphrodite gonads arise from pre-meiotic DNA damage). We have also included data (S1D Fig) specifically highlighting the difference between hermaphrodite and male SPO-11-independent COSA-1 foci.

10) The authors raise an interesting prior observation that “progeny sired by spo11(ok79) males have a higher viability than progeny derived from spo-11 mothers [40]” and raise the possibility that perhaps this improved fertility of spo-11 males is due to COSA-1 mediated CO formation. The rigorous experiment to address this question would be to remove COSA-1 from spo-11(ok79) males (i.e. create the cosa-1 spo-11 double mutant) and evaluate the viability of progeny sired. Instead, the viability of progeny sired by cosa-1 single mutant males is compared to the viability of progeny sired by spo-11 mutant males. A rigorous conclusion cannot be drawn about whether COSA-1 crossovers (or crossover-like entities) promote positive chromosome disjunction outcomes in spo-11 mutant males from the single mutant comparisons: There is the possibility that SPO-11-mediated (but COSA-1 independent) recombination intermediates promote positive chromosome disjunction outcomes in the cosa-1 single mutant and likewise that COSA-1 associated entities promote positive chromosome disjunction outcomes in the spo-11 single mutant (resulting in similar fertility between the two male genotypes). If the spo-11 cosa-1 double mutant cannot be analyzed (perhaps these genes are very tightly linked), the narrative should be revised to discuss these limitations to the interpretation.

We agree with the reviewer and tried to make the spo-11; cosa-1 double to examine male-sired progeny viability. Unfortunately, we were unsuccessful generating the strain, using two different approaches. While we think all the data are consistent with the absence of crossovers in the spo-11 mutant males (e.g., not dependent on RAD51, no change in progeny viability) we have rewritten this section as follows:

“To confirm that the COSA-1 foci observed in the absence of SPO-11 arise independently of HR and therefore do not lead to bona fide COs, we examined the viability of progeny sired by spo-11(ok79) or cosa-1(tm3298) mutant males. Note that in male worms, chromosome condensation at the end of meiotic prophase precludes analysis of bivalent formation as in hermaphrodites; progeny viability serves as an indirect readout of successful CO formation. As previously reported [43], 74.06±5.82% of progeny sired by spo-11 mutant males were inviable, presumably due to CO failure (S3D Fig). We observed a similar level of progeny inviability sired by cosa-1 males (74.68±3.08%; S3D Fig), consistent with an inability to form COs. Taken together, the lack of dependency on RAD-51 and the relatively high inviability of progeny sired by spo-11 males suggest that the COSA-1 foci recruited in the absence of SPO-11 do not represent interhomolog recombination events.”

11) (this is more of an aside): Could it be the case that spo-11 hermaphrodites cull germline nuclei with high numbers of COSA-1 foci? I seem to recall that spo-11 hermaphrodites display little germ cell apoptosis, but thought I should ask in case it is a possibility.

We think this is unlikely because, as indicated by the reviewer, germ cell apoptosis is lower in spo-11 mutants compared to wild type presumably because no breaks are made. Additionally, our analysis of COSA-1 foci formation in ced-3 mutants (Fig 1E), in which apoptosis is abrogated, revealed no statistical difference compared to WT, indicating that nuclei carrying extra COSA-1 foci are absent and not eliminated by apoptotic cell death.

12) Another suggestion for manuscript readability is to move the paragraph starting “To determine if other CO markers are also enriched with COSA-1 in the absence of SPO-11…” earlier, prior to the fertility data and RAD-51 co-localization / dependency experiments. These “COSA-1 partner” data naturally follow the original observation of COSA-1 foci, prior to conducting dependency experiments (which only analyze COSA1). I do think the RAD-51 colocalization and dependency experiments would be best presented as their own independent Results subsection.

We have changed as suggested by the reviewer.

13) I suggest limiting the interpretation of the SYP-2 experiments somewhat in the narrative (Results subtitle and some words throughout the manuscript that suggest SC is the only thing disrupted in a syp-2 mutant): If only SYP-2 is analyzed, it is most rigorous to conclude that a particular outcome reflects a role for SYP-2 (which may perhaps reflect a role for the SC). SYP-2 could possibly have an SC-independent role in promoting these lesions when SPO-11 is removed; more than one SC component should be analyzed if one wants to feel confident in attributing an outcome to lack of SC itself. So for example I suggest revising the Results subheading to “Sexual dimorphic role of SYP-2 in promoting and limiting recruitment of COSA-1 upon SPO11 depletion” (or “…SC component SYP-2…”) and revising sentence “We found that loss of synapsis significantly reduced the number of SPO-11-independent COSA-1 foci in males” to “We found that loss of SYP-2 significantly reduced the number of SPO-11-depletion-associated COSA-1 foci in males”, and the final sentence to “Altogether, our findings unveil a differential role for SYP-2 (and possibly the SC) in modulating the recruitment of…”

We have made these changes, as well as in the discussion, where we also state that this is likely a consequence of removing the SC.

14) “However, diakinesis nuclei of synapsis-defective mutants mostly contain achiasmatic univalent, suggesting that the residual COSA-1 is likely to be recruited at intermediates that do not originate from interhomolog recombination events.” Is there good evidence for this? Do COSA-1 foci in synapsis-defective germlines rely on RAD51? What about the possibility that COSA-1 foci in these synapsis-defective genotypes are associated with interhomolog recombination intermediates that become noncrossovers?

There are plenty of independent studies that show very little reduction in DAPI bodies number (which may possibly indicate formation of bivalents) in SYP mutants, which is not proportional to the number of COSA-1 foci detected in the germ lines of these mutants (more COSA-1 foci than putative bivalents in Diakinesis nuclei). The most likely scenario is that COSA-1 labels inter-sister recombination events, also considering that the homolog is not nearby in the absence of the SC and therefore only the invasion in the sister chromatid would be possible. It has been previously shown that in rec-8 mutants for example, in which the SC is highly aberrant, removal of COSA-1 results in complete separation of sister chromatids (Crawley et al.; 2016), indicating that indeed COSA-1 can be loaded at inter-sister recombination events.

15) Is it the case that irradiation of spo-11 males blocks the accumulation of late pachytene COSA-1 foci associated with SPO-11 removal? I believe this is the case, but the authors may want to bring this out in their discussion of pro-CO markers in late prophase nuclei being caused by “absence of DSBs per se rather than SPO-11 specifically”. The Discussion otherwise is thorough and helpful.

We have included this in the discussion.

Minor Comments:

16) The Introduction has a sentence that begins “COs shuffle maternal and paternal information and provide a physical connection between…” I suggest removing “ shuffle maternal and paternal information and” (mom and dad genetic information shuffles anyhow through independent assortment, and this study is about the more essential and cooler purpose of COs - physical connection, chromosome segregation, genome integrity).

This has been removed.

17) The DAPI signal throughout all figures should be changed (more cyan) to match that of Fig S2C.

We have kept the DAPI signal in blue, which is standard in the field.

18) The following sentences made me confused because I could not determine what the X chromosome had to do with interpreting the <5 foci result: “Compared to hermaphrodites [54, 55], a significant cohort of nuclei with <5 COSA-1 foci was observed in males (Fig. 6A). Since the single X chromosome present in males does not undergo CO recombination, this phenotype was somewhat surprising and…” I think it would be much clearer if “Since the single X chromosome present in males does not undergo CO recombination” was removed from the second sentence

Fixed.

19) “We noticed that under TOP-2 depletion conditions, the average number of COSA- foci was further enhanced compared to SPO-11 depletion only (Fig. 5A), which we partly attribute to the possible generation of polyploid cells arising from impaired cell division due to the loss of TOP-2.” This sentence should have a citation I think for the data showing that loss of TOP-2 leads to polyploid nuclei in the germline.

Done.

20) Author summary has “by the topoisomerase SPO-11…” the SPO-11 enzyme might not be considered a true topoisomerase by many since it doesn’t carry out the rejoining reaction on broken DNA– to avoid confusing the less specialized reader or irritating the more specialized reader may want to instead write “topoisomerase-like”.

Done.

21) Author summary could use a little revising to tell the reader that crossovers lead to physical attachments between chromosomes which are important for their proper segregation on the meiosis I spindle.

Done.

22) Figure 1 and Figure 2 legend titles refer to crossover numbers but measure COSA1 foci. I understand that the authors are using COSA-1 foci as a proxy for crossovers, but particularly in this case where the authors have some evidence for COSA-1 foci that do not correspond to a crossover intermediate it is more rigorous to state “does not alter COSA-1 foci numbers”.

Done.

---------------------

**Reviewer #2:**

1) Figure 3: While the difference in the kinetics of COSA-1 foci appearance following irradiation between males and hermaphrodites is clear, it may not be readily apparent to readers who are unfamiliar with the data presented in Yokoo et al., 2012.

As indicated in the response to Reviewer #1, we have included our own data of hermaphrodites treated with different doses of IR performed at the same time as the males. This is now presented in Fig 2.

2) Figure 4C: The % viable progeny sired by cosa-1 mutant males is comparable to that by spo-11 mutant males, supporting the conclusion that the improved viability by spo-11 males is not due to conversion of DSBs into crossovers. If this is not the cause, what alternative explanation might account for the increased viability?

This is included in the discussion:

“This likely reflects the operation of more efficient mechanism(s) for segregation of chromosomes lacking a CO (achiasmate chromosomes) in males compared to hermaphrodites [82]. Sex differences in the ability to segregate achiasmatic chromosomes may be attributable to differences in the organization of chromosomes within nuclei at the end of meiotic prophase [17], and/or differences in the organization and assembly of meiotic spindles [83, 84]. We also found previously that although checkpoints do not induce apoptosis in male germ cells, checkpoints are active and promote euploid sperm [21]. Alternatively, or in addition to achiasmatic chromosome segregation and robust checkpoint signaling, selection for high quality sperm may occur at fertilization.”

3) Figure 4A: Please provide a schematic showing how the regions (zones) are divided.

We have included a schematic representation of the germ line zoning as requested.

4) Figure 4B: I wonder whether there is a way to integrate the quantifications for RAD51 (4A) and COSA-1 (right panel) with the zoning information. Presenting both datasets on the same graph would clarify that COSA-1 foci form independently of RAD-51. Additionally, since RAD-51 and COSA-1 typically do not co-localize in wild type, the lack of co-localization may be expected and worth noting.

We could not integrate both quantifications in a single chart since the COSA-1 loading ensues after RAD-51 disengages from the chromatin, therefore the zoning between the two markers are different. In fact, the RAD-51 quantification was performed between transition zone throughout pachytene, whereas that COSA-1 quantification was assessed in the last 7 cell rows starting from the beginning of the condensation zone going backwards.

5) Page 14, In the final sentence starting with “This suggests that upon reduced physiological DSB levels”, an alternative explanation for the unaffected COSA-1 loading in him-5, him-17, and dsb-2 mutants is that the loss of these genes does not impair DSB formation in males to the same extent as in hermaphrodites. It would be important to demonstrate this by showing RAD-51 staining in these mutants.

We have included RAD-51 foci quantification in the male germ lines of the indicated mutants as requested (S4 Fig), which show that in him-5, him-17 and dsb-2 mutants, the reduction of RAD-51 foci number is not as extreme as observed in the hermaphrodite germ line, in particular for him-17 (Reddy and Villeneuve, 2004 – Janisiw et al., 2020) and dsb-2 mutants (Rosu et al.; 2013).

6) Page 15, the final sentence of the Results about the possibility of polyploid cells: is there any sign of polyploidy such as enlarged nuclei in the progenitor zone?

As also requested by the Editor, we have included representative images of the mitotic tip showing that under TOP-2 depletion, dramatic enlargement of mitotic cells is observed (S6B Fig).

7) Page 17, the first sentence: Cahoon et al., (2019 Genetics, Figure 2) reported that pro-CO factors such as MSH-5 and ZHP-3 form foci in spo-11; syp-1 and spo-11; syp2 mutant hermaphrodites. Thus, the observation of COSA-1 foci in males in the absence of SYP-2 and SPO-11 may not be entirely surprising. Although Cahoon et al. did not quantify these foci, their figure suggests that only a single focus is typically presented. This is very different from the broader distribution of COSA-1 foci shown in Figure 7B of the current manuscript. Could this be due to residual SPO-11 activity despite auxin treatment? Indeed, the authors discuss this possibility on page 21. It would be important to formally test this possibility using a genetic null.

The relevance of our analysis in the syp-2; spo-11::AID male germ line is represented by the fact that the COSA-1 accumulation triggered by loss of SPO-11 requires SYP2/SC. Regarding the surprising phenotype observed in the hermaphrodite gonad, we have generated the OLLAS::cosa-1; spo-11(ok79); syp-2(ok307) strain and analyzed COSA-1 foci as requested (S7 Fig). We found that COSA-1 accumulation in the double deletion mutant recapitulates that observed in the degron strain, indicating that this is not due to incomplete depletion. We believe that this might have been missed in Cahoon et al., since COSA-1 localizes in a much more robust way in late pachytene nuclei compared to GFP::MSH-5 or ZHP-3. Noteworthy in the latter, is that the overall levels of ZHP-3 in the absence of synapsis are greatly reduced, as also shown in the abovementioned manuscript cited by the Reviewer, where ZHP-3 signal in the spo-11; syp-1 is hardly detectable. Alternatively, although we think it is less likely, this phenotype could be specific to syp-2 mutation, since in Cahoon et al.; syp-1 mutant was employed.

---------------------

**Reviewer #3:**

1) In the shown examples of 6 COSA-1 foci, it seems that one of the foci is smaller than the others. Is this always the case? Could it be that this is on the X? Given that the Glo:COSA-1 is brighter than the previous transgenes, maybe this is recognizing an intersister exchange. Can staining with anti-HTP-3 show that the extra COSA-1 foci happen to be on a chromosome that already has another COSA-1 focus?

Tracing experiments are difficult to do in males as the chromosomes are more compact. However, our previous data suggest that the extra COSA-1 is not on the X chromosome (Checchi et al., 2014). Further, genetic mapping data indicate that there are double COs on autosomes in males, consistent with the extra COSA-1 foci marking COs on autosomes.

2) The problem with plotting the data as 5, 10, 50, 100Gy is that the dose is not linear, so actually the dose curve is asymptotic. The differences here could be due to eliciting different alternative repair pathways in the male germ line. IR breaks and SPO-11induced breaks are fundamentally different in their initial processing.

Thank you for pointing this out. We have remade the graph using a linear scale and have included hermaphrodite data (see response to Reviewers 1 and 2). This has allowed us to perform statistics to compare the male and hermaphrodite data.

3). p. 12. The statement “no colocalization with OLLAS::COSA-1, suggesting that chromatin recruitment of COSA-1 occurs independently of prior RAD-51 foci formation” is challenging. These two proteins do not colocalize in hermaphrodites either using traditional staining methods. RAD-51 is effectively replaced by MSH-5 and then COSA-1. That said, the following experiments showing lack of COSA-1 in the spo-11::AID rad-51 null do strongly support this conclusion, so perhaps just rephrase this sentence?

We have rephrased this sentence as follows: “We first immunolabeled RAD-51 in fixed OLLAS::cosa-1; spo-11::AID male germ lines from worms exposed to auxin for 24h and examined localization of RAD-51 and OLLAS::COSA-1. We observed very few RAD-51 foci upon SPO-11 depletion (Fig 4B), including in the region where

OLLAS::COSA-1 foci are observed, suggesting that chromatin recruitment of COSA-

1 occurs independently of prior RAD-51 foci formation.”

4). p. 13 spo-11::AID rad-51 TIR1::mRuby. This genotype is confusing – is TIR1 also on the same chromosome? What promoter is driving the TIR-1 here?

As indicated in the response to the Editor’s comments, we have replaced the inconsistent “TIR1” call with “ieSi38”, which corresponds to the original nomenclature adopted by the Dernburg lab, which generated this transgene (Zhang and Dernburg, 2015). Yes, ieSi38 encodes for Psun-1::TIR1::mRuby::sun-1 3’ UTR, which is linked to spo-11 and rad-51 loci on Chromosome IV.

5) It is not just that the COSA-1 foci are independent of SPO-11 but in the spo-11::AID on auxin, there is an altered distribution of COSA-1 foci. DO the authors have any thoughts or explanation for this? (this should also be mentioned specifically in the text).

We have included in the text that the foci have an altered distribution compared to “normal” COSA-1 foci and have also included a new S1D Figure, which emphasizes this point. We provide possible explanations for this observation in the discussion:

**“What is the nature of the COSA-1-marked sites in late meiotic prophase in spo11 mutants?**

Surprisingly we found that COSA-1 and associated partners are present and show an altered distribution at chromosomal sites at late meiotic prophase in spermatogenesis when SPO-11 is not present. We show that these “sites” are not dependent on other topoisomerases, nor HR, suggesting some type of chromosomal lesion or alteration is present in late pachytene/condensation zone that attracts these CO markers in male spermatocytes.” AND:

“One possibility to explain the presence of pro-CO markers at the end of meiotic prophase is that during the process of condensation, critical for packaging the genome into sperm, there are breaks or distortions in the DNA that provide sites for accumulation of COSA-1 and other CO markers. These lesions/sites do not accumulate COSA-1 in wild-type meiosis because COSA-1 is enriched at bona fide COs. Previous studies have shown that in meiotic mutants that perturb CO formation, COSA-1 can bind DNA at sites other than at COs [24, 33, 71, 74, 86]. An alternative possibility is that in the absence of SPO-11 chromatin changes could leave genomic sites vulnerable to accumulation of these pro-CO factors. The broad distribution of COSA-1 foci we observe, instead of the five (or six) that mark COs in wild type, is consistent with these marking some type of stochastic lesion or site.”

6) In figure 5C, please label not just GFP and OLLAS, but the specific protein to which they are attached. Also note that red-green colors are not color-blind friendly and change accordingly, if possible.

We have tried to include a color-blind friendly color code where possible. However, we have chosen to maintain the Blue/Green for DAPI/GFP::COSA-1 as this is the standard in the field.

7) The rightmost panel in Figure 5B appears to be mislabeled since there is not DNA stain there and it is unclear which foci these are.

We have fixed this (now Fig 4A).

8) Do RMH-1 foci show the same “extra” CO that were shown in Fig 1 for COSA-1?

We do observe more than 5 RMH-1 foci in some nuclei, as shown in Figu 4B.

9) It is unclear whether the proximal CO factor foci in the absence of SPO-11 are appearing AFTER late pachytene or still in LP?

They are overall present in late pachytene and all the way through the condensation zone, as we state in the text.

10) ATM-1 in hermaphrodite worms appears not to promote extra breaks as in mice (ref 75), although it is likely part of a feedback mechanism on DSBs.

This is published in Checchi et al., 2014 – there does appear to be more breaks as measured in the rad-54 background in male worms (Supp Figure 5).

11) Meneely show that Him-5 reduces the total number of DSBs in hermaphrodites including on autosomes. It is this feature which appears to be active in the males.

We agree that it is indeed the likely explanation to the phenomenon that we are observing, as we have also stated in the text.

12) Ref 10 does suggest that there can be 6 COSA-1 foci in the male germ line (see Fig 5, source data). Please reference this accordingly.

This reference has now been included.

13) “more RAD-51 foci are observed in males compared to hermaphrodite germ cells [10, 22, 23].” Ref 22 shows that number of RAD-51 foci in rad-54 mutants. Those numbers are close to published numbers for hermaphrodites. So I am not sure this is true.

We have reworded this to be more precise: there is an altered distribution.
